# Supplementary material for: Somatic driver mutation prevalence in 1844 prostate cancers identifies ZNRF3 loss as a predictor of metastatic relapse
Source: Nat Commun. 2021 Oct 29;12:6248. doi: 10.1038/s41467-021-26489-0 (PMC8556363; doi:10.1038/s41467-021-26489-0)
Supplement: Supplementary file 1 — Supplementary Figures and Tables [file 41467_2021_26489_MOESM1_ESM.pdf]

Supplementary Figures and Tables

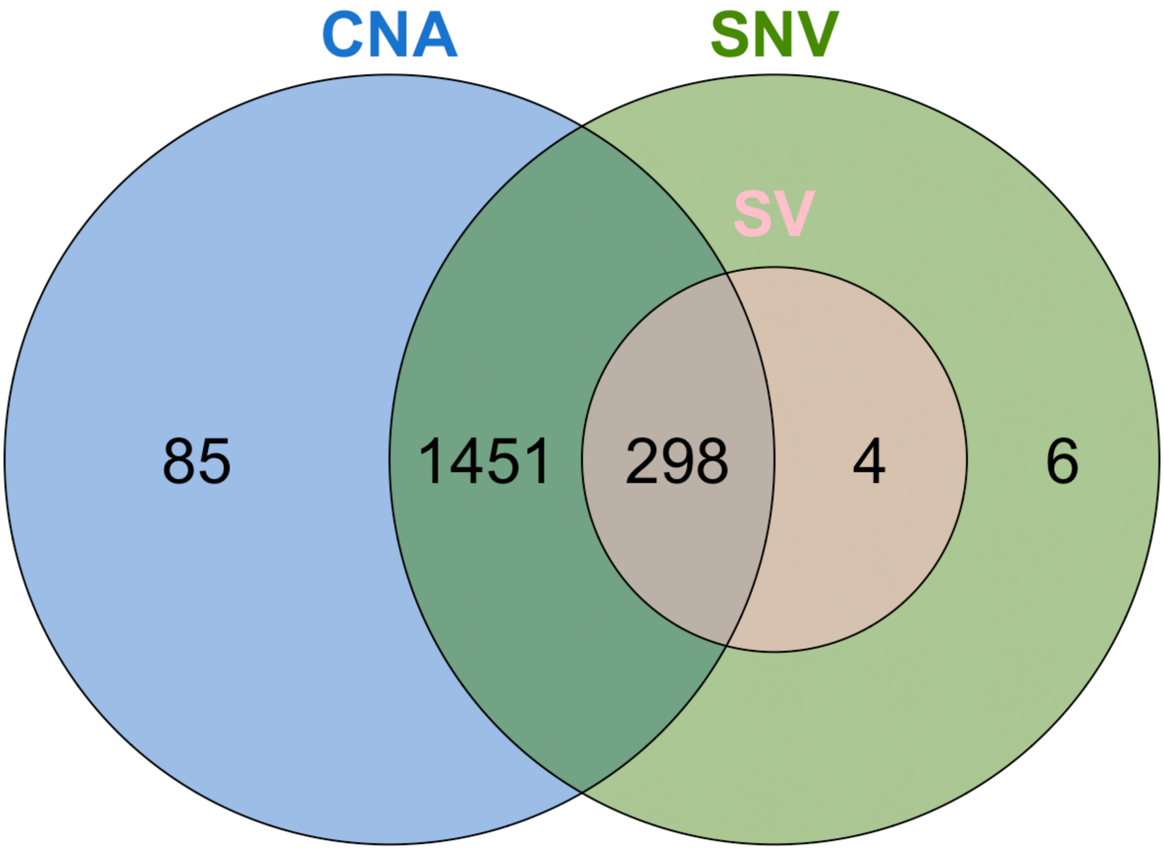

Figure S1 – Data Availability for Localized Prostate Cancer Cases

Overlap of available samples for SNV, CNA, and SV data from 1,844 patients in the study cohort. Colours represent mutation type; blue: CNA, green: SNV, taupe: SV.

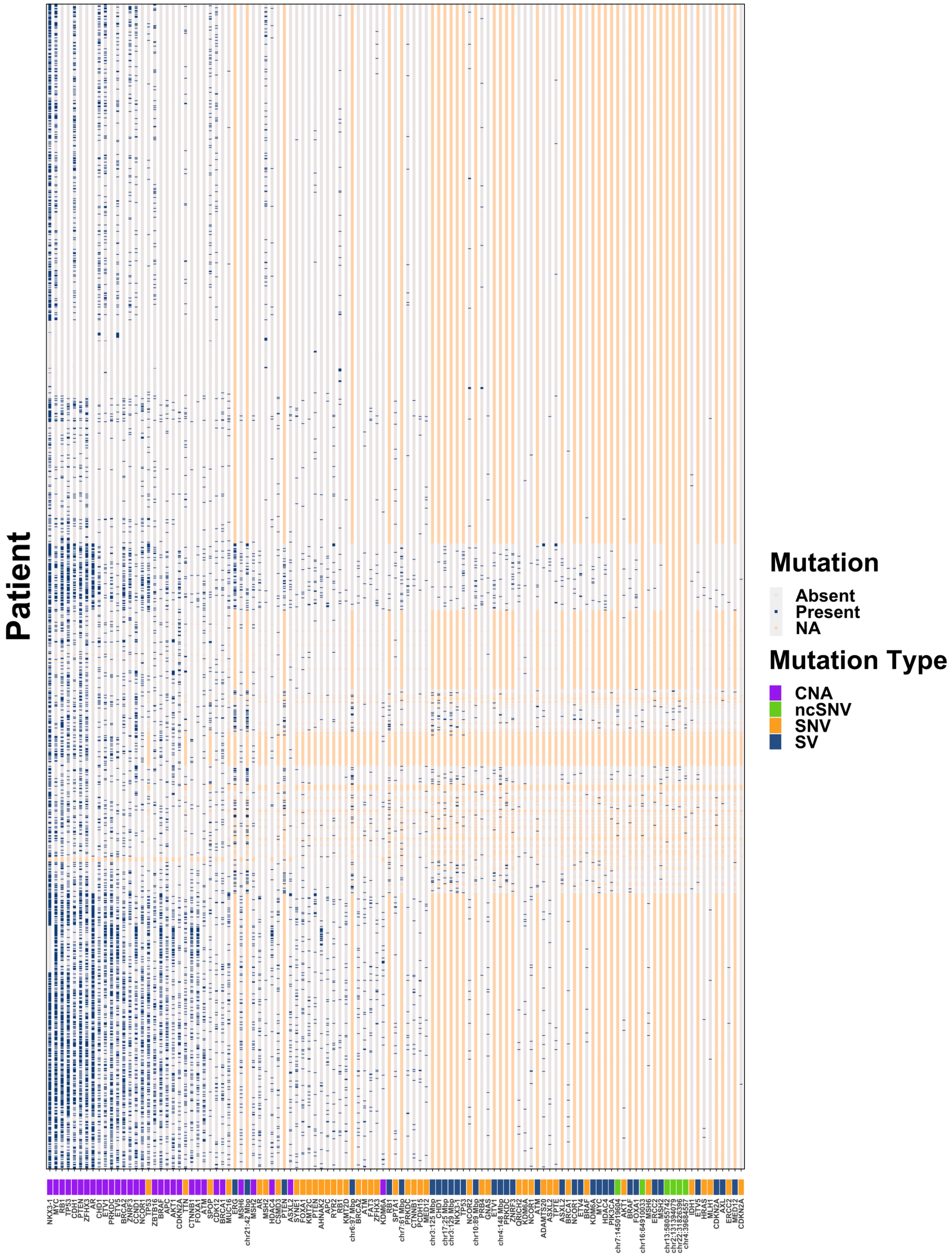
Patient
Mutation
Absent
Present
NA
Mutation Type
CNA
ncSNV
SNV
SV

Figure S2 – Heatmap of Driver Gene Mutations in 1,884 Prostate Cancers

Each row represents an individual patient. Each column is a separate driver gene mutation. Blue and white represent the presence or absence of the specific mutation in the particular patient, respectively. Light brown indicates that this mutation type was not analyzed in the specific patient. Mutation type covariates: purple: CNA, green: non-coding SNV, orange: SNV, blue: SV.

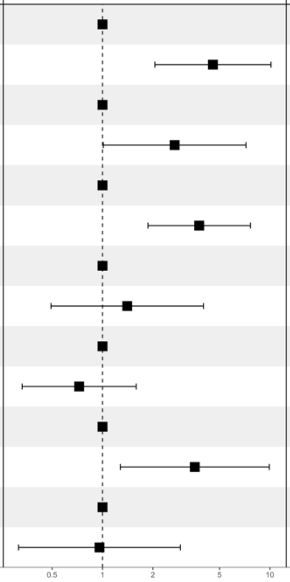

Variable		N	Hazard ratio		p
**ZNRF3**	Neutral	349		Reference	
	Loss	27		4.56 (2.05, 10.14)	<0.001
**MYC**	Neutral	309		Reference	
	Gain	67		2.70 (1.01, 7.19)	0.048
**CCND1**	Neutral	296		Reference	
	Gain	80		3.78 (1.87, 7.65)	<0.001
**PRKDC**	Neutral	328		Reference	
	Gain	48		1.40 (0.49, 4.01)	0.525
**TP53**	Neutral	287		Reference	
	Loss	89		0.73 (0.33, 1.59)	0.422
**CDK12**	Neutral	358		Reference	
	Loss	18		3.56 (1.28, 9.91)	0.015
**ETV5**	Neutral	348		Reference	
	Loss	28		0.96 (0.32, 2.92)	0.942

Figure S3 – Multivariable Cox Proportional Hazards Model of Prognostic, mCRPC-Enriched Driver Mutations

Association between risk of metastatic relapse in men with localized prostate cancer (CPCG; n = 376) and CNAs in *MYC*, *CCND1*, *PRKDC* (gain), and *ZNRF3*, *TP53*, *ETV5*, and *CDK12* (loss) were assessed in a multivariable Cox proportional hazards model including all four of these driver CNAs. *ZNRF3*, *MYC*, *CCND1*, and *CDK12* remained independently prognostic on multivariable analysis. Error bars represent 95% confidence intervals of the reported hazard ratios. Two-sided p-values generated from a Wald test. ZNRF3: $p = 1.94 \times 10^{-4}$; CCND1: $p = 2.18 \times 10^{-4}$

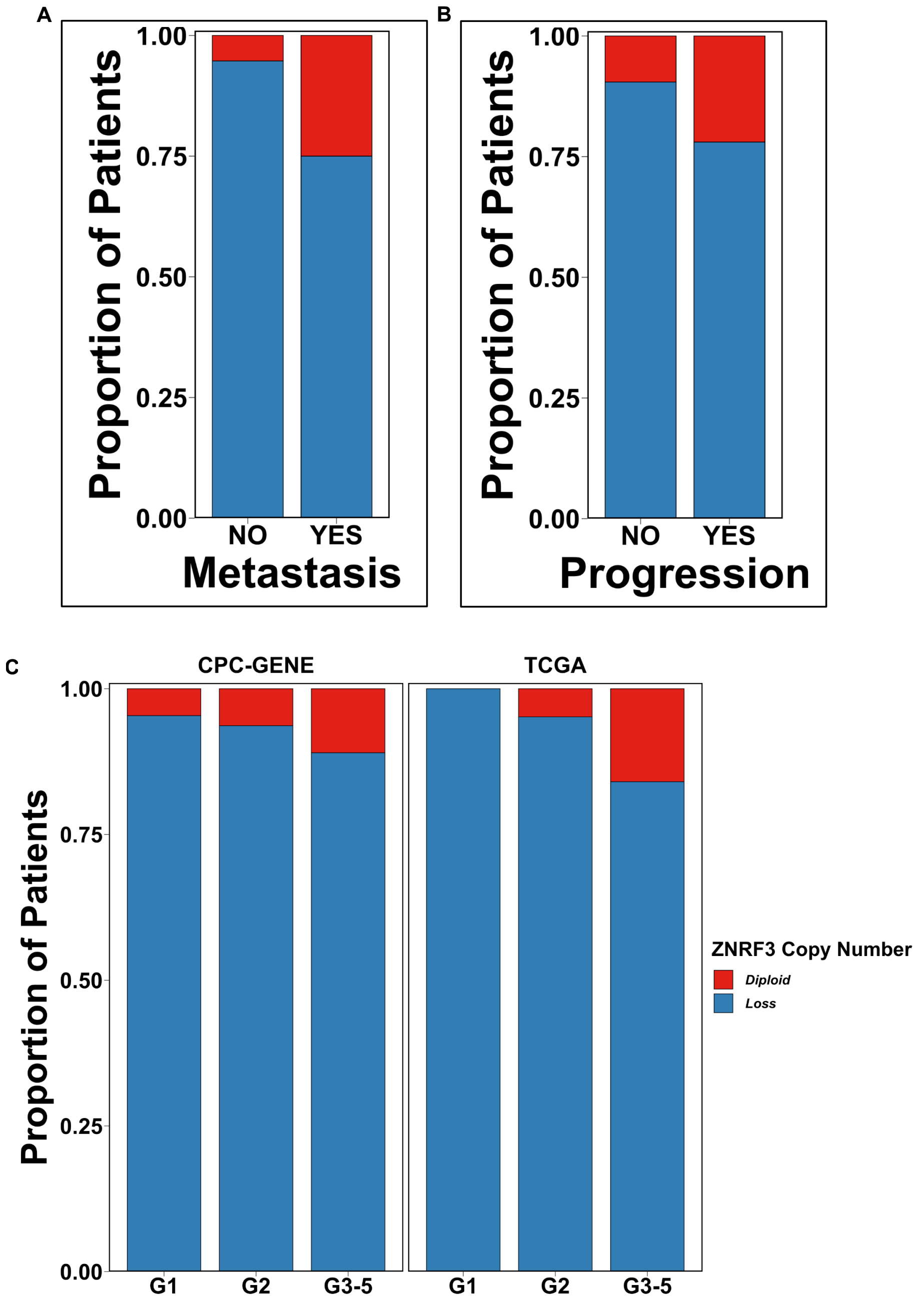

Figure S4 – Frequency of ZNRF3 loss in Localized Prostate Cancers, Stratified by Metastasis or Progression

Patients were stratified based on whether they experienced a metastatic relapse (A; CPCG cohort) or disease progression (B; TCGA cohort). C. Proportion of cases in CPCG and TCGA harbouring ZNRF3 loss, stratified by ISUP grade (G1: Grade 1; G2: Grade 2; G3-5: Grades 3-5). Blue indicates the proportion of patients who are *ZNRF3* neutral; red indicates patients with *ZNRF3* loss.

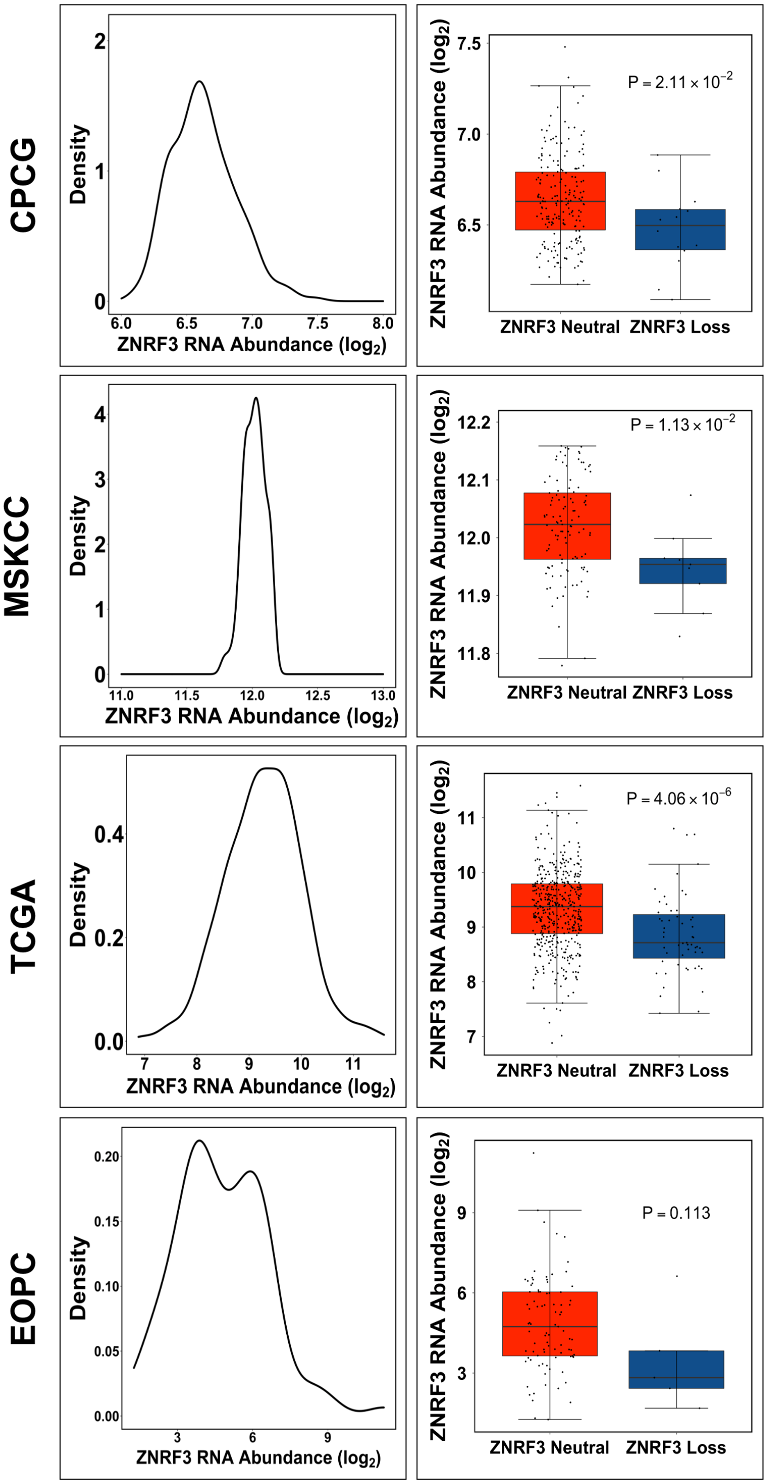

Figure S5 – ZNRF3 Loss is Associated with Reduced ZNRF3 RNA Abundance

ZNRF3 RNA abundance was assessed in four independent cohorts (CPCG: n = 208; MSKCC; n = 131; TCGA: n = 493; EOPC: n = 118, as indicated) and stratified based on ZNRF3 loss. Left panels show density plots of $\log_2$ ZNRF3 RNA abundance in each cohort. Right panels show $\log_2$ ZNRF3 RNA abundance stratified by *ZNRF3* loss. P-values are from a Mann-Whitney U test. Centre of box represents the median value. Lower and upper box hinges correspond to the first and third quartile. Whiskers extend to the largest and smallest values no further than 1.5-times the Interquartile Range.

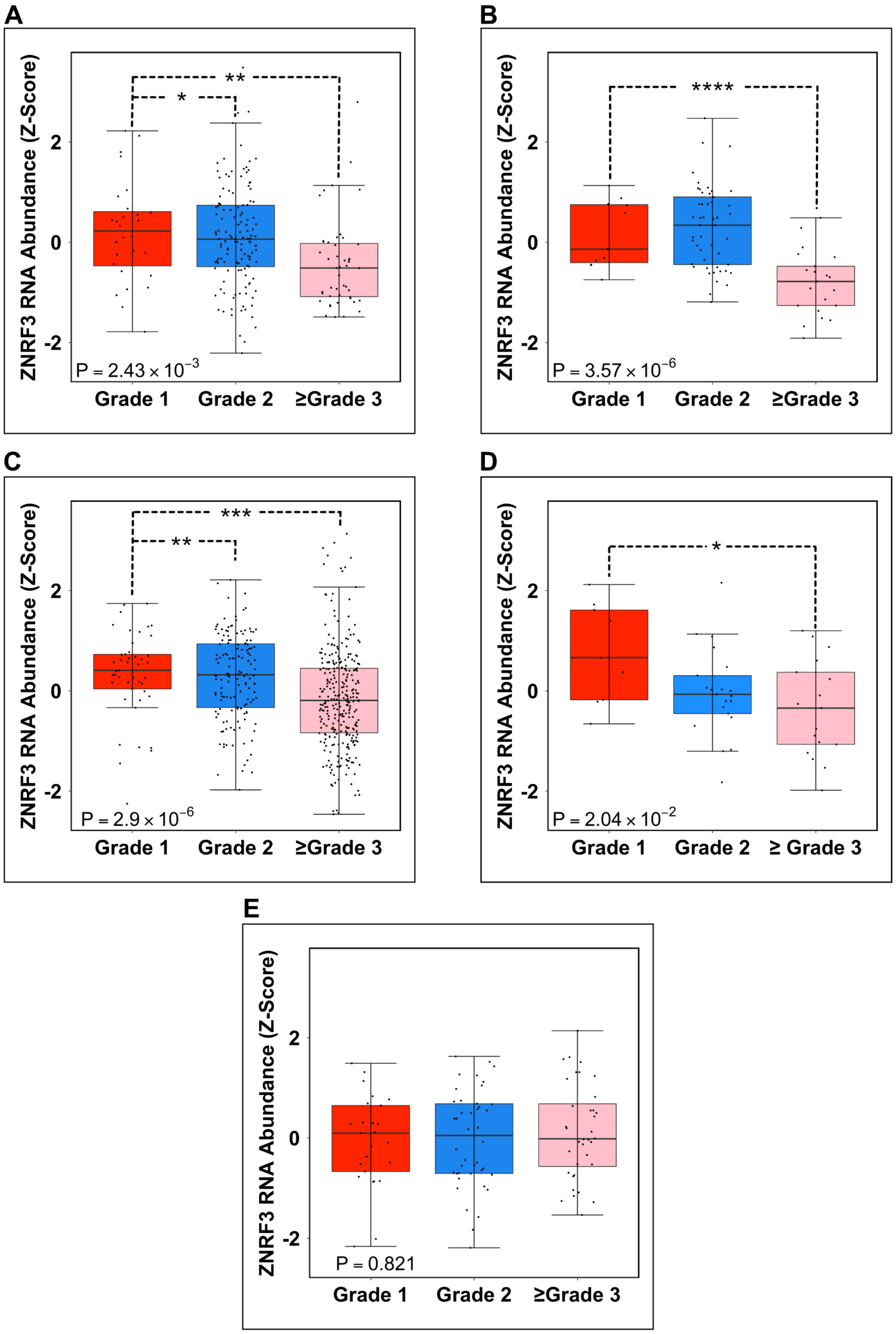

Figure S6 – ZNRF3 RNA Abundance is Inversely Associated with Tumor ISUP Grade

Associations between diagnostic ISUP grade and ZNRF3 RNA abundance were assessed for four independent cohorts (A: CPCG, B: EOPC, C: TCGA, D: LTRI, E: MSKCC). P-value shown is from a one-way Analysis of Variance with Tukey post-hoc tests to assess between-groups significance, indicated as follows: **** - $p < 0.0001$; *** - $p < 0.001$; ** - $p < 0.01$; * - $p < 0.05$. Centre of box represents the median. Lower and upper box hinges correspond to the first and third quartile. Whiskers extend to the largest and smallest values no further than 1.5-times the Interquartile Range. A: **: $p = 0.004$; *: $p = 0.02$. B: ****: $p = 1.9 \times 10^{-6}$. C: ***: $p = 4.13 \times 10^{-5}$; **: $p = 3.52 \times 10^{-3}$; D: *: $p = 0.015$.

A

Variable	Level	N	Hazard Ratio	HR (95% CI)	p-value
**ZNRF3 RNA Abundance**		205		0.22 (0.06, 0.87)	0.03
**ISUP Grade**	*1*	28		Reference	
	2	131		2.07 (0.61, 7.06)	0.25
	3	46		3.01 (0.83, 10.86)	0.09
**PSA**	*<10ng/ml*	159		Reference	
		46		1.24 (0.60, 2.56)	0.55
**Clinical T-Category**	*cT1*	112		Reference	
	cT2a/b	88		1.68 (0.92, 3.06)	0.09
	cT2c	5		0.99 (0.13, 7.49)	0.99

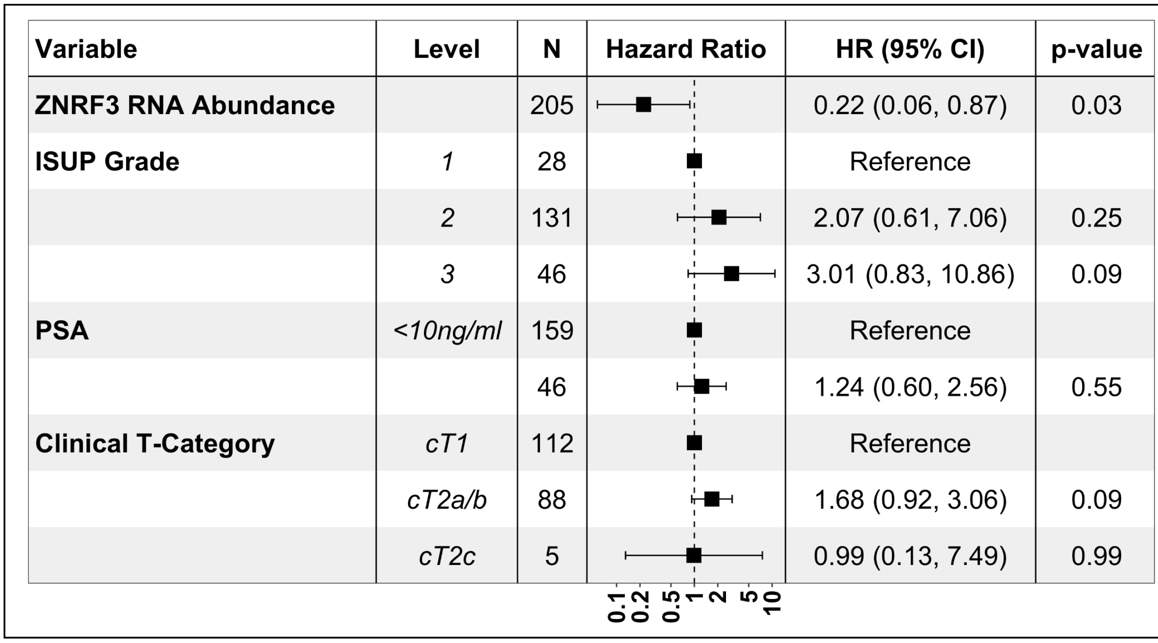

B

Variable	Level	N	Hazard Ratio	HR (95% CI)	p-value
**ZNRF3 RNA Abundance**		205		0.21 (0.05, 0.78)	0.02
**ZNRF3**	*Neutral*	191		Reference	
	Loss	14		1.93 (0.80, 4.68)	0.14

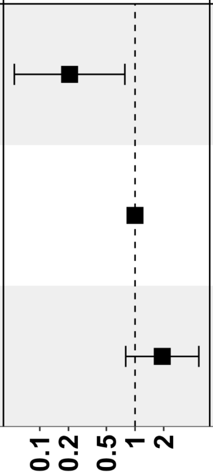

Figure S7 – ZNRF3 RNA Abundance is Inversely Associated with Risk of Metastatic Relapse

Multivariable Cox proportional hazards model of A. ZNRF3 RNA abundance (continuous) and clinical prognostic factors on biochemical relapse or B. ZNRF3 RNA abundance and *ZNRF3* genomic loss. P-values from a Wald test. Error bars represent 95% confidence intervals of the reported hazard ratios.

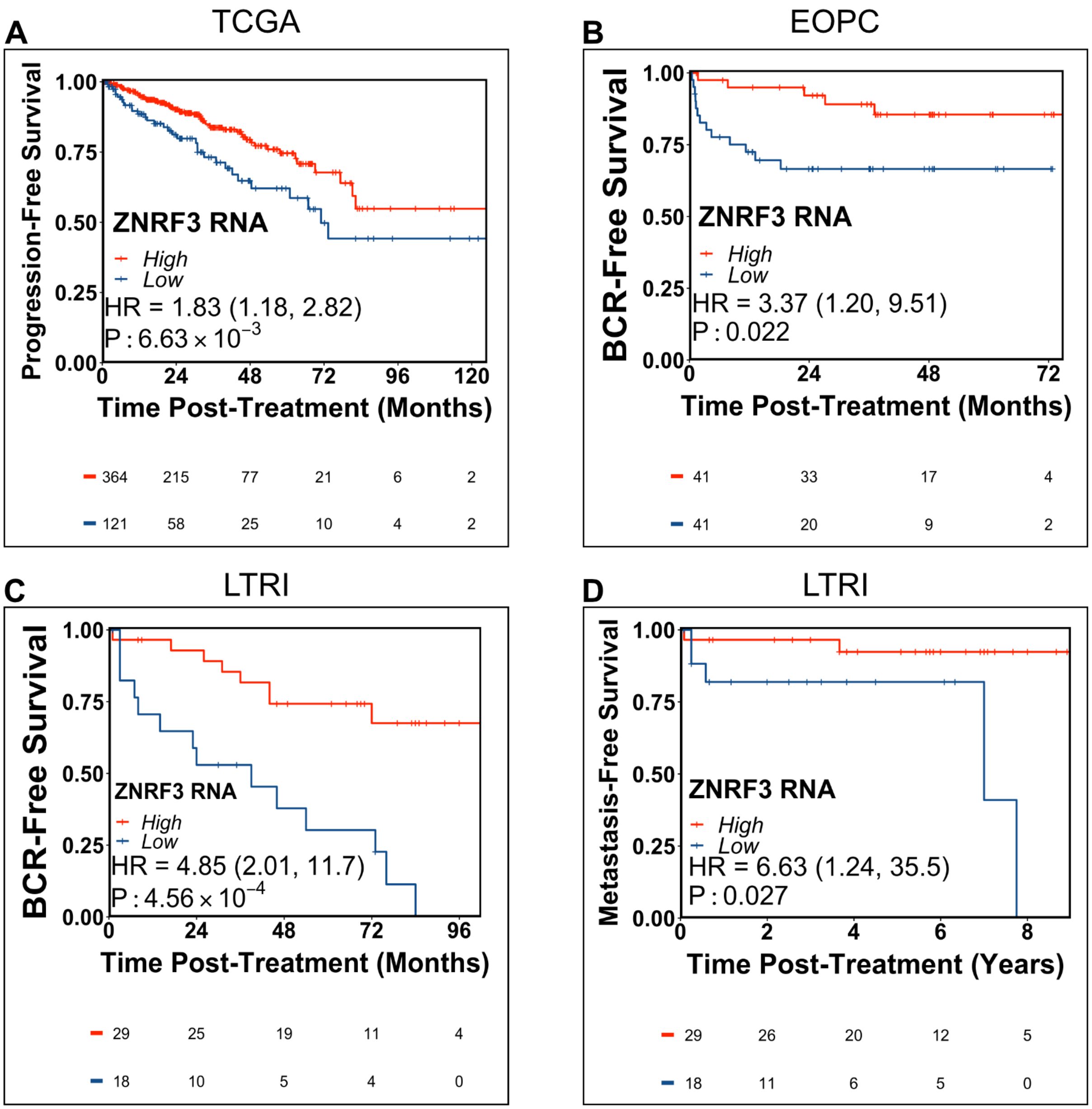

Figure S8 – Validation of Association of Low ZNRF3 RNA Abundance and Poor Clinical Outcome in Localized Prostate Cancer

Low ZNRF3 RNA abundance was associated with risk of progression-free survival (A; TCGA), biochemical relapse in the EOPC (B) and LTRI (C) cohorts, and metastatic relapse in the LTRI cohort (D). Two-sided p-values were generated from a Wald test.

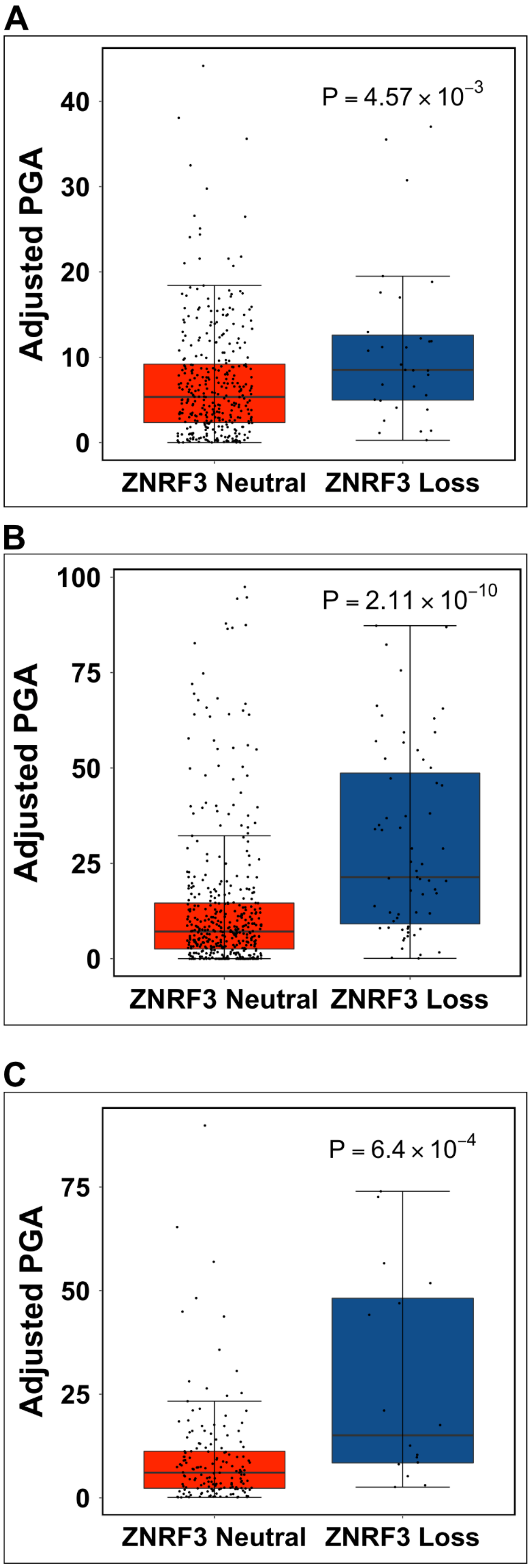

Figure S9 – ZNRF3 Loss is Associated with Increased Genomic Instability

Patients in the CPCG (A; n = 380), TCGA (B; n = 493), or MSKCC (C; n = 174) cohorts were stratified by ZNRF3 copy number status. Adjusted PGA was calculated as the total number of bases affected by a CNA divided by the total number of bases in the genome, excluding chromosome 22 in both cases. Two-sided p-values were generated from a Mann-Whitney U test. Centre of box represents the median value. Lower and upper box hinges correspond to the first and third quartile. Whiskers extend to the largest and smallest values no further than 1.5-times the Interquartile Range.

Variable	Level	N	Hazard Ratio	HR (95% CI)	p-value (Wald test)
**ZNRF3**	*Neutral*	298		Reference	
	Loss	28		3.02 (1.24, 7.36)	0.015
**ISUP Grade**	*1*	54		Reference	
	2	190		3.43 (0.43, 27.37)	0.244
	3	73		7.77 (0.96, 63.09)	0.055
	4	9		3.84 (0.23, 65.02)	0.352
**PSA**		326		1.03 (0.94, 1.13)	0.503
**Clinical T-Category**	*cT1*	157		Reference	
	cT2a/b	160		1.62 (0.67, 3.91)	0.283
	cT2c	9		2.25 (0.26, 19.67)	0.464
**Adjusted PGA**		326		1.05 (1.02, 1.09)	0.006
**IDC-P/CA**	*Absent*	222		Reference	
	Present	104		1.57 (0.72, 3.43)	0.257

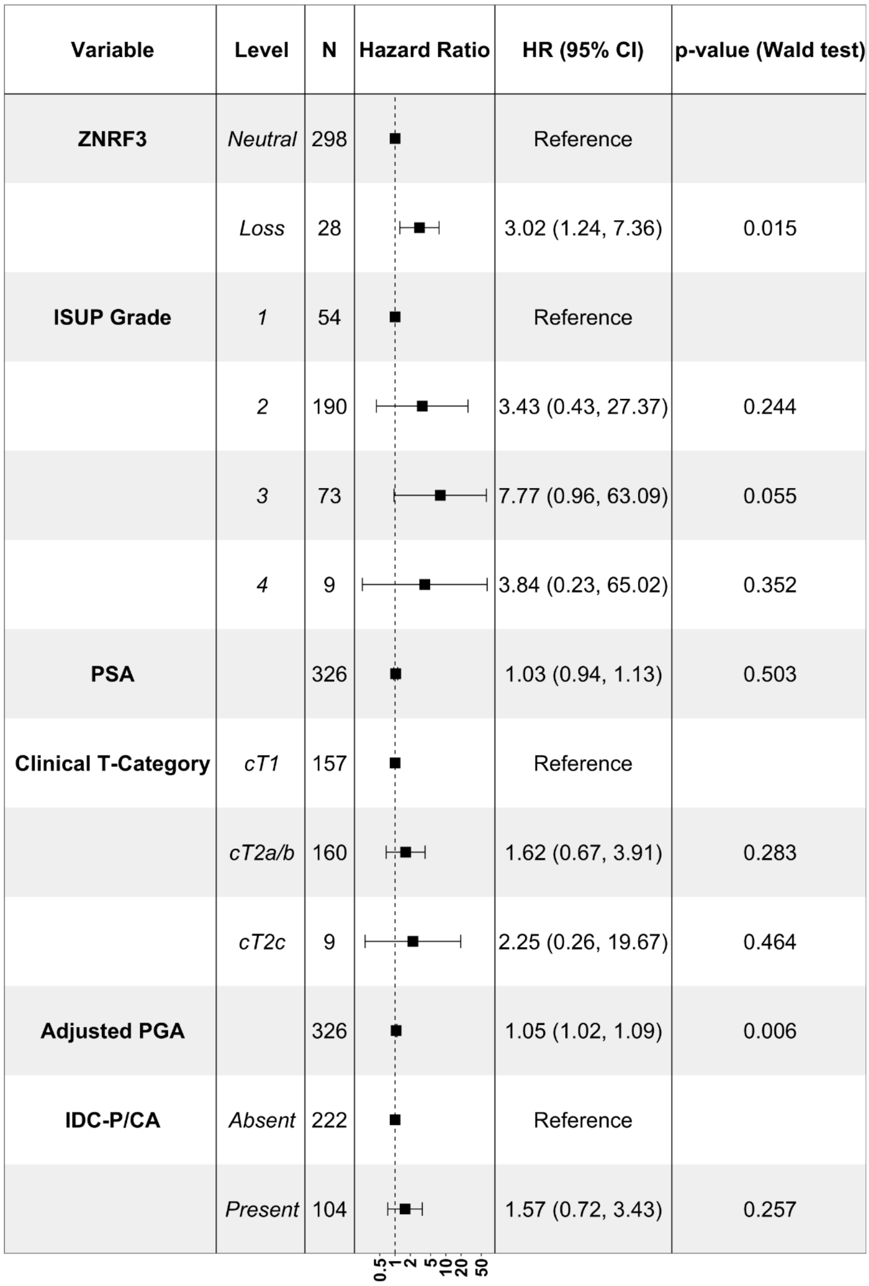

Figure S10 – ZNRF3 Loss is an Independent Prognostic Factor for Metastatic Relapse in Localized Prostate Cancer

Multivariable Cox proportional hazards model (metastatic relapse) of *ZNRF3* loss with ISUP grade, pre-treatment PSA, clinical T-category, adjusted PGA, and the presence of intraductal carcinoma of the prostate or cribriform architecture (IDC-P/CA). Error bars represent 95% confidence intervals of the reported hazard ratios.

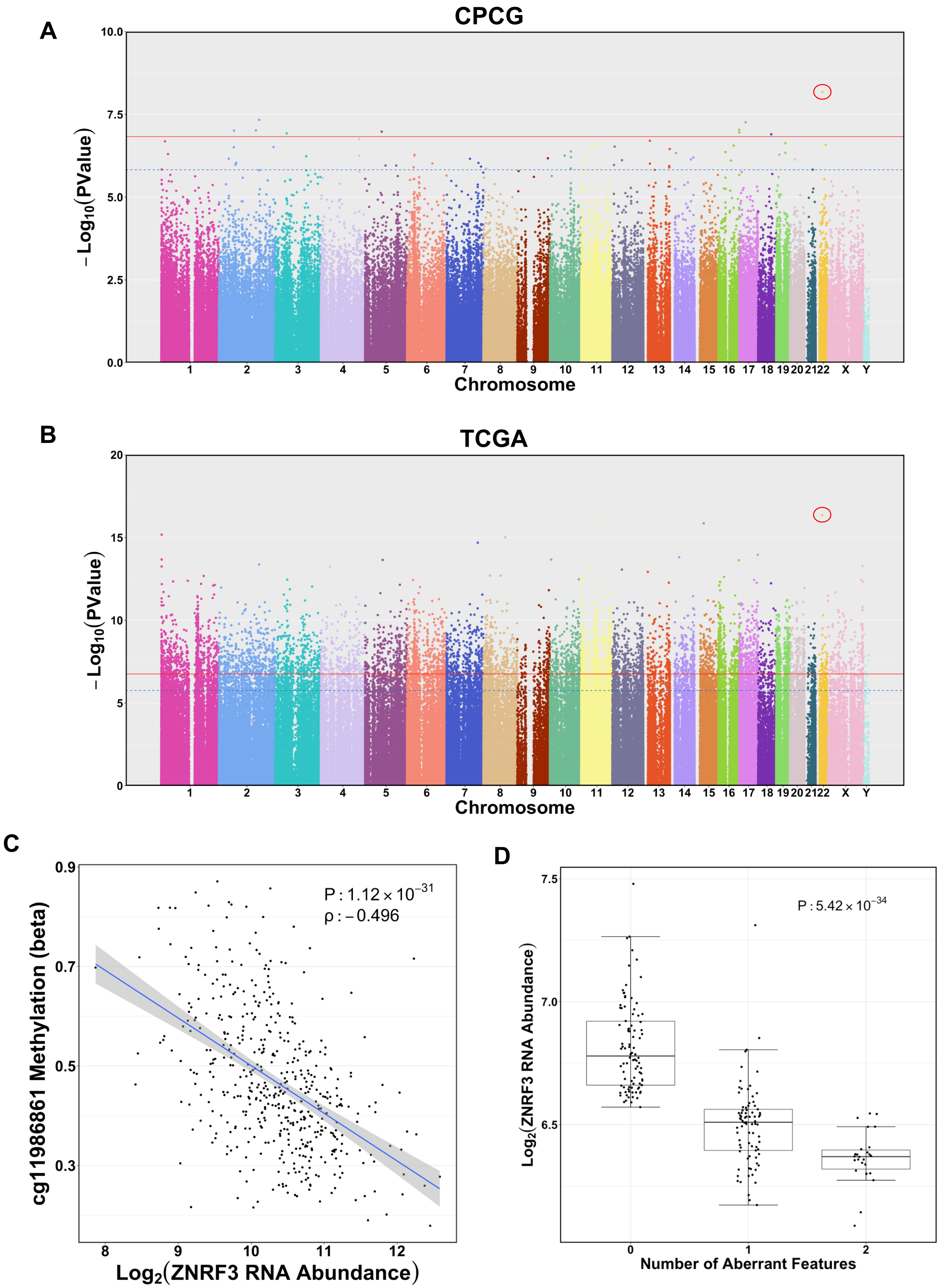

A
CPCG
−Log10(PValue)
Chromosome
B
TCGA
−Log10(PValue)
Chromosome
C
P : 1.12 × 10^−31
ρ : −0.496
cg11986861 Methylation (beta)
Log2(ZNRF3 RNA Abundance)
D
P : 5.42 × 10^−34
Log2(ZNRF3 RNA Abundance)
Number of Aberrant Features

Figure S11 – Differential DNA methylation in tumours based on ZNRF3 RNA abundance

A. & B. Differential DNA methylation analysis in CPCG (A) and TCGA (B), using ZNRF3 RNA abundance to dichotomize patients into high or low bins. Red line shows genome-wide error rate cutoff using a Bonferroni correction; dashed blue line shows suggestive significance (*i.e.* 1/10 Bonferroni). C. Inverse correlation between ZNRF3 RNA abundance and ZNRF3 5' promoter methylation in TCGA. Shaded area shows the 95% confidence interval of the best fit line. D. Patients harbouring both *ZNRF3* deletion and promoter hypermethylation (n = 26) have significantly lower ZNRF3 RNA abundance than those harbouring either deletion or hypermethylation alone (n = 81) or harbouring neither alteration (n = 94). Two-sided p-values were generated from a Kruskal-Wallis test. Centre of box represents the median value. Lower and upper box hinges correspond to the first and third quartile. Whiskers extend to the largest and smallest values no further than 1.5-times the Interquartile Range.

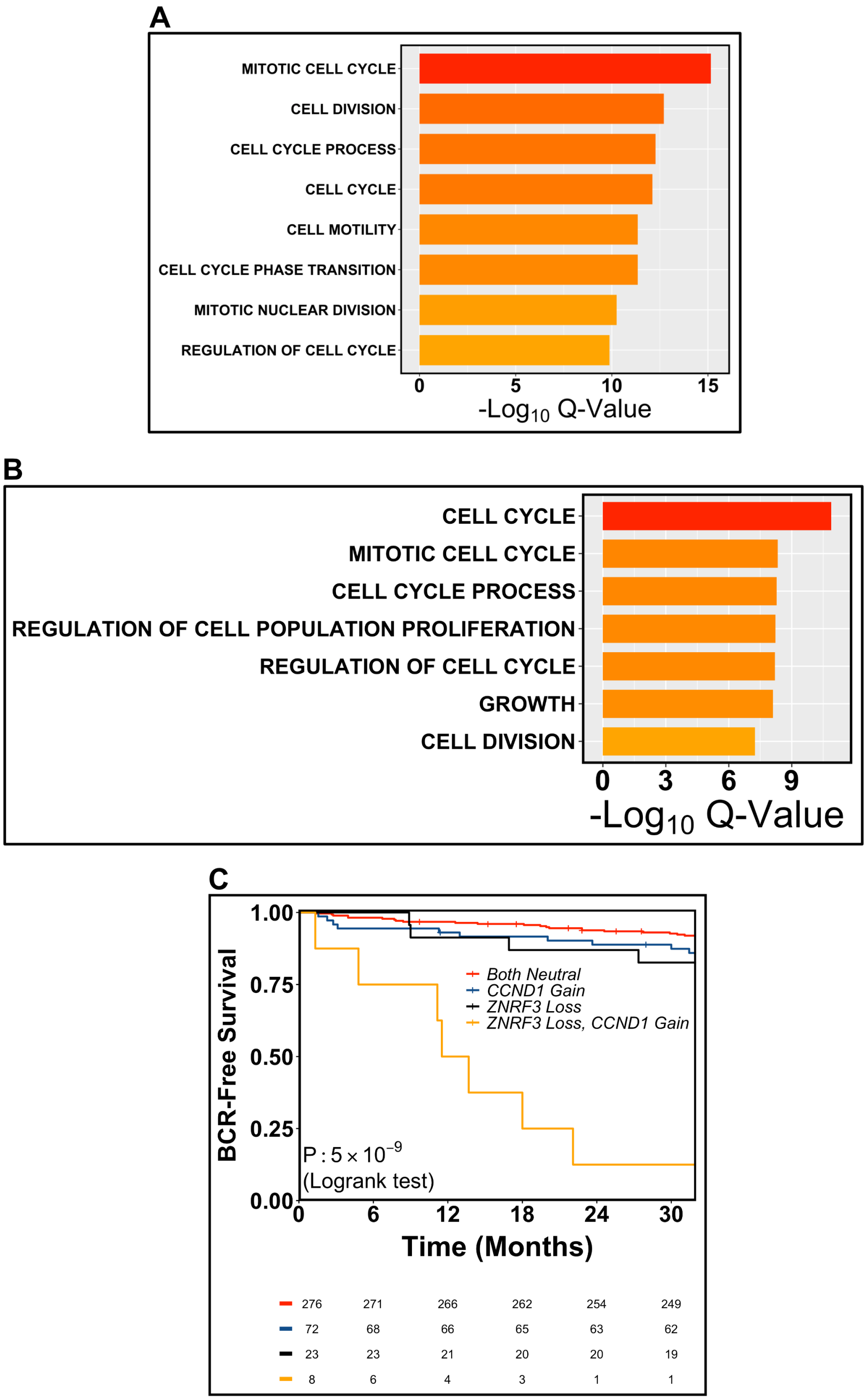

Figure S12 – Associations Between ZNRF3 Loss and Cell Cycle Progression

Gene Set Enrichment Analysis of tumors harboring *ZNRF3* loss in the EOPC cohort of localized prostate cancer (A) and the Abida cohort of mCRPC (B). C. Biochemical relapse-free survival in CPCG patients, stratified by *ZNRF3* loss and *CCND1* gain.

Supplementary Tables

Gene.Locus	type	HR	HR.Upper	HR.Lower	p.value	q.value
MYC	CNA	4.23	2.2	8.3	2.70E-05	6.00E-04
CCND1	CNA	3.89	2	7.7	9.70E-05	7.60E-04
ZNRF3	CNA	4.58	2.1	9.9	1.00E-04	7.67E-04
PRKDC	CNA	3.33	1.6	7	0.0014	8.05E-03
CDK12	CNA	3.31	8.54	1.28	1.34E-02	0.062
ETV5	CNA	2.37	0.92	6.1	0.075	0.25
TP53	CNA	1.84	0.94	3.6	0.076	0.25
ETV1	CNA	2.01	0.83	4.9	0.12	0.307
CHD1	CNA	1.92	0.87	4.2	0.11	0.307
ZFHX3	CNA	1.59	0.8	3.2	0.19	0.437
PTEN	CNA	1.47	0.75	2.9	0.27	0.565
ZBTB16	CNA	1.56	0.55	4.4	0.4	0.767
BRCA2	CNA	1.41	0.55	3.7	0.47	0.772
CDH1	CNA	0.82	0.32	2.1	0.69	0.934
RB1	CNA	0.89	0.43	1.9	0.78	0.953
NKX3-1	CNA	1.01	0.52	2	0.99	1
SPOP	SNV	1.1	0.33	3.7	0.87	0.953
MSH2	SNV	NA	NA	NA	NA	NA
chr17: 25Mbp	SV	1.73	0.39	7.7	0.47	0.772
ERG	SV	0.8	0.28	2.3	0.68	0.934
chr21:42Mbp	SV	0.62	0.082	4.8	0.65	0.934
ETV1	SV	1.23	0.16	9.4	0.84	0.953
chr10: 89Mbp	SV	0.86	0.24	3.1	0.83	0.953
TP53	SV	1.34	0	Inf	1	1

Table S1 - Univariable Cox Proportional Hazards Modeling (Metastasis-Free Survival) of Driver Mutations Enriched in mCRPC and Present in at Least 5% of Localized Cancers. Two-sided p-values were generated from a Wald test. Q-values were calculated using the Benjamini-Hochberg FDR method.

CNA	Univariable HR (95% CI)	Univariable Q-Value	Multivariable HR (95% CI)	Multivariable Q-Value
MYC Gain	1.53 (1.01 - 2.32)	4.72E-02	1.56 (0.833 - 3.06)	1.59E-01
CCND1 Gain	1.84 (1.04 - 3.25)	0.0361	1.20 (0.629 - 2.28)	0.582
PRKDC Gain	1.26 (0.80 - 1.99)	0.319	0.786 (0.402 - 1.54)	0.483
ZNRF3 Loss	2.56 (1.56 - 4.21)	2.06E-04	2.34 (1.39 - 3.94)	1.41E-03

Table S2 - Progression-Free Survival in TCGA patients, Stratified by Driver CNAs. Two-sided p-values were generated from a Wald test. Q-values were calculated using the Benjamini-Hochberg FDR method.

CNA	HR (95% CI)	Q-Value	Test
MYC Gain	12.4 (4.03 - 38.0)	3.18E-05	CoxPH; Wald
CCND1 Gain	11.1 (2.42 - 50.8)	1.94E-03	CoxPH; Wald
PRKDC Gain	11.1 (3.72 - 33.2)	3.18E-05	CoxPH; Wald
ZNRF3 Loss	7.67 (2.52 - 23.3)	4.40E-04	CoxPH; Wald

Table S3 - Biochemical Relapse in Taylor/MSKCC cohort patients, Stratified by Driver CNAs. Two-sided p-values were generated from a Wald test. Q-values were calculated using the Benjamini-Hochberg FDR method.

CNA	OR (95% CI)	Q-Value	Test
MYC Gain	4.46 (1.98 - 10.0)	0.00132	Fisher's Exact test
CCND1 Gain	16.2 (1.84 - 143)	0.00456	Fisher's Exact test
PRKDC Gain	4.48 (1.82 - 11.0)	0.0016	Fisher's Exact test
ZNRF3 Loss	5.78 (2.07 - 16.1)	0.0016	Fisher's Exact test

Table S4 - Associations Between Driver CNAs and Metastatic Relapse in Taylor cohort patients. Two-sided p-values were generated from a Fisher's Exact test. Q-values were calculated using the Benjamini-Hochberg FDR method.

Factor	Level	Number of Patients	HR	95% CI	p-value (Wald Test)
ISUP Grade	1	65	Reference	-	-
	2	221	6.04	0.809 - 45.2	0.08
	3	80	17	2.21 - 131	0.0065
	4 and above	10	6.04	0.371 - 98.5	0.207
Clinical T-Category	cT1	179	Reference	-	-
	cT2a/b	187	2.25	1.07 - 4.70	0.032
	cT2c	10	2.5	0.318 - 19.6	0.384
PSA (Continuous)	Continuous	376	1.06	1.01 - 1.12	0.014
PSA (Discrete)	<10ng/ml	274	Reference		
	≥10ng/ml	102	2.07	1.06 - 4.04	0.034

Table S5 - Univariable Cox Proportional Hazards Models of Clinical Prognostic Factors on Metastatic Relapse, CPC-GENE Cohort. Two-sided p-values were generated from a Wald test.

				Biochemical Relapse	
Variable	Level	N	HR	95% CI	P-Value
Fraser Signature	Continuous	375	1.59	1.37 - 1.85	1.29E-09
ZNRF3 CNA					
	Diploid	344	-	-	-
	Loss	31	1.77	1.06 - 2.98	3.00E-02
PSA	Continuous	375	1.04	1.00 - 1.07	0.031
ISUP Grade					
	1	65	-	-	-
	2	180	0.81	0.48 - 1.36	0.42
	3 and above	90	1.43	0.82 - 2.49	0.209

				Metastatic Relapse	
Variable	Level	N	HR	95% CI	P-Value
Fraser Signature	Continuous	375	1.58	1.17 - 2.12	2.69E-03
ZNRF3 CNA					
	Diploid	344	-	-	-
	Loss	31	2.86	1.29 - 6.34	0.019
PSA	Continuous	375	1.07	0.999 - 1.15	0.055
ISUP Grade					
	1	65	-	-	-
	2	180	5.95	0.761 - 46.6	0.089
	3 and above	90	13.5	1.72 - 106	0.014

Table S6 - Multivariable Cox Proportional Hazards Analysis of ZNRF3 Loss and the 6-Feature Fraser Signature effects on Biochemical and Metastatic Relapse, controlled for ISUP grade, PSA, and clinical T category. Two-sided p-values were generated from a Wald test.

symbol	ID	Chr	Start	End
LIMK2	NM_016733;NM_005569	22	31608249	31676066
KREMEN1	NM_001039570;NM_032045	22	29469065	29564321
ASCC2	NM_001242906	22	30184596	30234293
HSCB	NM_172002	22	29138042	29153496
RNF185	NM_152267	22	31556137	31603005
EWSR1	NM_005243	22	29663997	29696515
ZNRF3	NM_001206998	22	29279754	29453476
XBP1	NM_005080	22	29190547	29196560
CCDC117	NM_173510	22	29168661	29185283
RHBDD3	NM_012265	22	29655843	29663914
APOBEC3D	NM_152426	22	39417117	39429256
UQCR10	NM_013387	22	30163357	30166402
MTMR3	NM_153051	22	30279157	30426857
HORMAD2	NM_152510	22	30476452	30573062
CHEK2	NM_001257387	22	29083730	29137822
TTC28	NM_001145418	22	28374001	29075853
ZMAT5	NM_019103	22	30126944	30162969
EMID1	NM_001267895	22	29601900	29655586
ZNRF3.AS1	NR_046851	22	29420986	29427464
NIPSNAP1	NM_003634	22	29950797	29977326
APOBEC3C	NM_014508	22	39410264	39414825
APOBEC3F	NM_145298	22	39436672	39451975
APOBEC3G	NM_021822	22	39473009	39483748
MIR3928	NR_037496	22	31556047	31556105
APOBEC3H	NM_001166002	22	39493228	39500072
APOBEC3A	NM_001270406	22	39353526	39359188
C22orf31	NM_015370	22	29454659	29457907
CABP7	NM_182527	22	30116343	30127820
SYNGR1	NM_004711	22	39745953	39781593

symbol	HR	CI Lower	CI Upper	p.value
LIMK2	5.6	2.6	12	1.30E-05
KREMEN1	5.9	2.9	12	1.30E-06
ASCC2	5.3	2.5	11	1.20E-05
HSCB	5.9	3	12	5.20E-07
RNF185	5.6	2.6	12	1.30E-05
EWSR1	5.6	2.7	11	2.80E-06
ZNRF3	5.9	3	12	5.20E-07
XBP1	5.9	3	12	5.20E-07
CCDC117	5.9	3	12	5.20E-07
RHBDD3	5.6	2.7	11	2.80E-06
APOBEC3D	6.8	2.9	16	1.50E-05
UQCR10	5.3	2.5	11	1.20E-05
MTMR3	5.3	2.5	11	1.20E-05
HORMAD2	5.3	2.5	11	1.20E-05
CHEK2	5.9	3	12	5.20E-07
TTC28	5.6	2.7	11	2.50E-06
ZMAT5	5.3	2.5	11	1.20E-05
EMID1	5.6	2.7	11	2.80E-06
ZNRF3.AS1	5.6	2.7	11	2.80E-06
NIPSNAP1	5.3	2.5	11	1.20E-05
APOBEC3C	6.8	2.9	16	1.50E-05
APOBEC3F	6.8	2.9	16	1.50E-05
APOBEC3G	6.8	2.9	16	1.50E-05
MIR3928	5.6	2.6	12	1.30E-05
APOBEC3H	6.8	2.9	16	1.50E-05
APOBEC3A	6.8	2.9	16	1.50E-05
C22orf31	5.9	2.9	12	1.30E-06
CABP7	5.3	2.5	11	1.20E-05
SYNGR1	6.8	2.9	16	1.50E-05

symbol	rank	adj.p.val	log.q.val	rna.p.val	rna.p.val.rank	rna.q.val
LIMK2	20	7.11E-03	2.1481	5.87E-05	1	1.70E-03
KREMEN1	6	7.11E-04	3.1481	0.0004974	2	7.21E-03
ASCC2	13	6.56E-03	2.1828	0.0008887	3	8.59E-03
HSCB	1	2.84E-04	3.5460	0.003864	5	2.24E-02
RNF185	20	7.11E-03	2.1481	0.00358	4	2.60E-02
EWSR1	9	1.53E-03	2.8149	0.005435	6	2.63E-02
ZNRF3	1	2.84E-04	3.5460	0.007748	7	3.21E-02
XBP1	1	2.84E-04	3.5460	0.009518	8	3.45E-02
CCDC117	1	2.84E-04	3.5460	0.01219	9	3.93E-02
RHBDD3	9	1.53E-03	2.8149	0.02532	10	7.34E-02
APOBEC3D	23	8.21E-03	2.0859	0.05507	13	1.23E-01
UQCR10	13	6.56E-03	2.1828	0.04945	11	1.30E-01
MTMR3	13	6.56E-03	2.1828	0.05493	12	1.33E-01
HORMAD2	13	6.56E-03	2.1828	0.08418	15	1.63E-01
CHEK2	1	2.84E-04	3.5460	0.08056	14	1.67E-01
TTC28	8	1.37E-03	2.8641	0.1268	16	2.30E-01
ZMAT5	13	6.56E-03	2.1828	0.1815	17	3.10E-01
EMID1	9	1.53E-03	2.8149	0.2435	18	3.92E-01
ZNRF3.AS1	9	1.53E-03	2.8149	0.3257	20	4.72E-01
NIPSNAP1	13	6.56E-03	2.1828	0.3163	19	4.83E-01
APOBEC3C	23	8.21E-03	2.0859	0.3541	21	4.89E-01
APOBEC3F	23	8.21E-03	2.0859	0.4053	23	5.11E-01
APOBEC3G	23	8.21E-03	2.0859	0.3986	22	5.25E-01
MIR3928	20	7.11E-03	2.1481	0.4548	24	5.50E-01
APOBEC3H	23	8.21E-03	2.0859	0.5277	25	6.12E-01
APOBEC3A	23	8.21E-03	2.0859	0.6666	26	7.44E-01
C22orf31	6	7.11E-04	3.1481	0.7213	27	7.75E-01
CABP7	13	6.56E-03	2.1828	0.8033	28	8.32E-01
SYNGR1	23	8.21E-03	2.0859	0.8641	29	8.64E-01

Table S7 - Survival Analysis (Metastasis) for Genes Showing Co-Deletion With ZNRF3 In At Least One CPC-GENE Case and Association with Metastatic Relapse Two-sided p-values for survival analyses were generated from a Wald test. Two-sided p-values for RNA-CNA concordance were generated from a Mann-Whitney U test. Q-values were calculated using the Benjamini-Hochberg FDR method.

CPCG - Metastasis

Gene	HR	Upper 95% CI	Lower 95% CI	P-Value	Q-Value
LIMK2	0.685	2.49	0.076	0.566	0.566
KREMEN1	0.252	0.833	0.076	0.0238	0.0428
ASCC2	0.276	1.09	0.0698	0.0665	0.0998
HSCB	0.233	0.739	0.0735	0.0133	0.0428
RNF185	0.377	1.15	0.1239	0.0855	0.11
EWSR1	0.371	1.33	0.103	0.128	0.144
ZNRF3	0.198	0.731	0.0538	0.0151	0.0428
XBP1	0.376	0.719	0.197	3.11E-03	2.80E-02
CCDC117	0.296	0.823	0.107	0.0196	0.0428

TCGA - Progression

Gene	HR	Upper 95% CI	Lower 95% CI	P-Value	Q-Value
LIMK2	0.888	1.09	0.727	0.248	0.279
KREMEN1	0.734	0.951	0.567	0.0192	0.0347
ASCC2	0.863	1.06	0.704	0.157	0.202
HSCB	1.06	1.3	0.866	0.569	0.569
RNF185	0.517	0.651	0.41	2.20E-08	1.98E-07
EWSR1	1.4	1.72	1.15	9.00E-04	0.00405
ZNRF3	0.736	0.952	0.569	0.0194	0.0347
XBP1	0.747	0.961	0.581	2.31E-02	3.47E-02
CCDC117	0.754	0.958	0.594	0.0207	0.0347

EOPC - Biochemical Relapse

Gene	HR	Upper 95% CI	Lower 95% CI	P-Value	Q-Value
LIMK2	0.863	1	0.746	0.0492	0.11
KREMEN1	0.636	1.01	0.567	0.0613	0.11
ASCC2	0.979	1.08	0.887	0.675	0.675
HSCB	0.906	1.02	0.805	0.105	0.158
RNF185	0.517	0.651	0.41	1.13E-05	5.09E-05
EWSR1	0.921	1.04	0.813	1.98E-01	0.255
ZNRF3	0.436	0.61	0.311	1.27E-06	1.14E-05
XBP1	1	1	0.994	2.86E-01	3.22E-01
CCDC117	0.858	0.992	0.741	0.0384	0.11

LTRI - Metastasis

Gene	HR	Upper 95% CI	Lower 95% CI	P-Value	Q-Value
LIMK2	0.527	1.11	0.252	0.09	0.203
KREMEN1	0.204	0.689	0.06	0.011	0.05
ASCC2	0.838	2.04	0.344	0.698	0.788
HSCB	0.875	2.32	0.33	0.788	0.788
RNF185	0.488	2.82	0.0844	1.16E-01	2.09E-01
EWSR1	0.762	1.62	0.358	4.80E-01	0.72
ZNRF3	0.176	0.525	0.059	0.00184	0.0166
XBP1	0.557	0.993	0.312	4.70E-02	1.41E-01
CCDC117	0.871	1.81	0.42	0.71	0.788

Table S8 - Survival Analysis with RNA Abundance of Genes Co-Deleted With ZNRF3, Across Four Independent Cohorts. Two-sided p-values were generated from a Wald test. Q-values were calculated using the Benjamini-Hochberg FDR method.

gene.id	gene.symbol	log2.fold.control	p.value	log10.pval
83999	KREMEN1	-0.313872701	0.000160569	3.794337324
2487	FRZB	0.793632518	0.001175909	2.929626134
84133	ZNRF3	-0.223141772	0.00454452	2.342511966
1277	COL1A1	0.478023086	0.008539946	2.068544885
26585	GREM1	0.425329395	0.009207131	2.035875659
51176	LEF1	0.545516237	0.011949741	1.922641505
10213	PSMD14	0.339816566	0.016075454	1.793836762
23240	TMEM131L	0.324279969	0.038010658	1.420094609
55897	MESP1	-0.203779227	0.038596721	1.413449591
5682	PSMA1	0.271003506	0.046609444	1.331526074
6424	SFRP4	0.547582972	0.048122167	1.317654828
56998	CTNNBIP1	-0.116363238	0.049359412	1.306630025

Table S9 - WNT Pathway Genes Differentially Abundant in CPC-GENE and TCGA Cases with ZNRF3 Loss. Two-sided p-values were generated using Student's t-tests, using empirical Bayes pooled variances.

			TCGA			CPCG	
Gene	Level	N	HR (95% CI)	P-Value	N	HR (95% CI)	P-Value
ZNRF3	Neutral	431	-	-	349	-	-
	Loss	58	2.35 (1.41 - 3.92)	1.08E-03	27	5.47 (2.53 - 11.8)	1.45E-05
APC	Neutral	422	-	-	345	-	-
	Loss	67	1.97 (1.19 - 3.25)	8.15E-03	34	1.32 (0.464 - 3.73)	0.606
CTNNB1	Neutral	449	-	-	369	-	-
	Loss	40	1.18 (0.597 - 2.31)	0.641	11	0 (0 - Inf)	1

Table S10 - Multivariable Cox Proportional Hazards Models of ZNRF3, APC, and CTNNB1 in TCGA and CPC-GENE. Two-sided p-values were generated from a Wald test. Q-values were calculated using the Benjamini-Hochberg FDR method.
