# Supplementary material for: Somatic driver mutation prevalence in 1844 prostate cancers identifies ZNRF3 loss as a predictor of metastatic relapse
Source: Nat Commun. 2021 Oct 29;12:6248. doi: 10.1038/s41467-021-26489-0 (PMC8556363; doi:10.1038/s41467-021-26489-0)
Supplement: Supplementary file 4 — Reporting Summary [file 41467_2021_26489_MOESM4_ESM.pdf]

Corresponding author(s): Michael Fraser, Ph.D
Paul C. Boutros, MBA, Ph.D

Last updated by author(s): Sep 28, 2021

Reporting Summary

Nature Portfolio wishes to improve the reproducibility of the work that we publish. This form provides structure for consistency and transparency in reporting. For further information on Nature Portfolio policies, see our Editorial Policies and the Editorial Policy Checklist.

Statistics

For all statistical analyses, confirm that the following items are present in the figure legend, table legend, main text, or Methods section.

n/a	Confirmed	
☐	☒	The exact sample size (n) for each experimental group/condition, given as a discrete number and unit of measurement
☐	☒	A statement on whether measurements were taken from distinct samples or whether the same sample was measured repeatedly
☐	☒	The statistical test(s) used AND whether they are one- or two-sided<br>*Only common tests should be described solely by name; describe more complex techniques in the Methods section.*
☐	☒	A description of all covariates tested
☐	☒	A description of any assumptions or corrections, such as tests of normality and adjustment for multiple comparisons
☐	☒	A full description of the statistical parameters including central tendency (e.g. means) or other basic estimates (e.g. regression coefficient) AND variation (e.g. standard deviation) or associated estimates of uncertainty (e.g. confidence intervals)
☐	☒	For null hypothesis testing, the test statistic (e.g. F, t, r) with confidence intervals, effect sizes, degrees of freedom and P value noted<br>*Give P values as exact values whenever suitable.*
☒	☐	For Bayesian analysis, information on the choice of priors and Markov chain Monte Carlo settings
☒	☐	For hierarchical and complex designs, identification of the appropriate level for tests and full reporting of outcomes
☐	☒	Estimates of effect sizes (e.g. Cohen's d, Pearson's r), indicating how they were calculated

Our web collection on statistics for biologists contains articles on many of the points above.

Software and code

Policy information about availability of computer code

Data collection	CGDS-R (v1.3.0)
Data analysis	R (v3.5.2), BoutrosLab.plotting.general (v5.9.2), VennDiagram (v1.6.20), survival (v2.43-3), ggplot2 (v3.1.0), easyGgplot2 (v1.0), survminer (v0.4.3), forestplot (v1.7.2), factorial2x2 (v0.2.0), cowplot (v1.1.0), tidyverse (v1.2.1), limma (v3.13), lumi (v2.24.0), cutpointr (v1.1.1), STAR (v2.5.2a), EdgeR (v3.12.1), mSigDB (v7.0), gGOSt (version e99_eg46_p14_f929183d)<br><br>Custom code is available from our GitHub repository at https://github.com/mfraser3/ZNRF3_2021 and from Zenodo at https://doi.org/10.5281/zenodo.5389194

For manuscripts utilizing custom algorithms or software that are central to the research but not yet described in published literature, software must be made available to editors and reviewers. We strongly encourage code deposition in a community repository (e.g. GitHub). See the Nature Portfolio guidelines for submitting code & software for further information.

Data

Policy information about availability of data

All manuscripts must include a data availability statement. This statement should provide the following information, where applicable:

- Accession codes, unique identifiers, or web links for publicly available datasets
- A description of any restrictions on data availability
- For clinical datasets or third party data, please ensure that the statement adheres to our policy

The genomic variant calls, clinical, and pathology data for the CPC-GENE study are available through the ICGC Data Portal under accession PRAD-CA [https://dcc.icgc.org/projects/PRAD-CA]. Raw whole genome sequencing data for the CPC-GENE study are available through the European Genome-Phenome Archive used

accession ID EGAD00001003761 [https://ega-archive.org/datasets/EGAD00001003761]. Raw DNA methylation data are available from the NCBI Gene Expression Omnibus under accession number GSE84043 [https://www.ncbi.nlm.nih.gov/geo/query/acc.cgi?acc=GSE84043]. Summary DNA methylation data are available from Figshare (https://doi.org/10.6084/m9.figshare.16574486.v1). Raw and processed mRNA array data for the CPC-GENE study are available from the NCBI Expression Omnibus under accession number GSE107299 [https://www.ncbi.nlm.nih.gov/geo/query/acc.cgi?acc=GSE107299]. Genomic variant calls, mRNA abundance, clinical, and pathology data are available from cBioPortal for the Abida (accession prad_su2c_2019) [https://www.cbioportal.org/study/summary?id=prad_su2c_2019], Barbieri (accession prad_broad) [https://www.cbioportal.org/study/summary?id=prad_broad], Baca (accession prad_broad_2013) [https://www.cbioportal.org/study/summary?id=prad_broad_2013], Robinson (accession prad_su2c_2015) [https://www.cbioportal.org/study/summary?id=prad_su2c_2015], Taylor (accession prad_mskcc) [https://www.cbioportal.org/study?id=prad_mskcc], Gerhauser and Weischenfeldt studies (accession prostate_dkfz_2018) [https://www.cbioportal.org/study/summary?id=prostate_dkfz_2018]. Raw sequencing data, genomic variant calls, mRNA abundance, and DNA methylation data for the TCGA study are available from cBioPortal (accession prad_tcga_pan_can_atlas_2018) [https://www.cbioportal.org/study/summary?id=prad_tcga_pan_can_atlas_2018] and the GDC Data Portal (accession TCGA-PRAD) [https://portal.gdc.cancer.gov/projects/TCGA-PRAD]. Processed genomic variant, mRNA abundance, and clinical/pathology data for the Quigley study are available from the authors' website (https://quigleylab.ucsf.edu/data). Processed mRNA abundance, and clinical/pathology data for the LTRI cohort are available from Zenodo (https://doi.org/10.5281/zenodo.5389194). Patients in the LTRI cohort were not specifically consented for deposition of raw sequencing data into public repositories. Therefore, raw RNAseq data for the LTRI cohort are available upon request to Dr. Michael Fraser and are subject the requestor entering into a Data Sharing Agreement with the Lunenfeld-Tanenbaum Research Institute. Raw data must not be shared outside of the requestor's research team, unless specifically authorized by the Lunenfeld-Tanenbaum Research Institute. The requestor must attest that no attempt will be made to identify any individual(s) in the cohort based on data provided. We will attempt to make data available within one month of any request.
Source data are provided as a Source Data file.

Field-specific reporting

Please select the one below that is the best fit for your research. If you are not sure, read the appropriate sections before making your selection.

☒ Life sciences ☐ Behavioural & social sciences ☐ Ecological, evolutionary & environmental sciences

For a reference copy of the document with all sections, see nature.com/documents/nr-reporting-summary-flat.pdf

Life sciences study design

All studies must disclose on these points even when the disclosure is negative.

Sample size	Sample sizes were based on available data
Data exclusions	Patients were only excluded based on lack of available data (e.g. RNA abundance, outcome data, etc).
Replication	Replication was achieved through validation in multiple cohorts. ZNRF3 CNA data were validated in four cohorts (CPCG, TCGA, MSKCC, EOPC). ZNRF3 RNA data were validated across five cohorts (CPCG, TCGA, MSKCC, EOPC, LTRI). ZNRF3 methylation data were validated in two cohorts (CPCG, TCGA). Pathological associations were validated in CPCG, TCGA, EOPC, and LTRI.<br><br>Gene expression associated with ZNRF3 CNA or loss was validated in CPCG, TCGA, and the Abida mCRPC cohorts.
Randomization	Not applicable to this observational study.
Blinding	Not applicable to this observational study.

Reporting for specific materials, systems and methods

We require information from authors about some types of materials, experimental systems and methods used in many studies. Here, indicate whether each material, system or method listed is relevant to your study. If you are not sure if a list item applies to your research, read the appropriate section before selecting a response.

Materials & experimental systems

n/a	Involved in the study	
☒	☐	Antibodies
☒	☐	Eukaryotic cell lines
☒	☐	Palaeontology and archaeology
☒	☐	Animals and other organisms
☐	☒	Human research participants
☒	☐	Clinical data
☒	☐	Dual use research of concern

Methods

n/a	Involved in the study	
☒	☐	ChIP-seq
☒	☐	Flow cytometry
☒	☐	MRI-based neuroimaging

Human research participants

Policy information about studies involving human research participants

Population characteristics	Patients were men with localized or metastatic, castration-resistant prostate cancer. No other inclusion or exclusion criteria were used, beyond whatever was employed in the source study.<br><br>CPCG focused on intermediate risk prostate cancer, irrespective of age. Patients were treated with radical prostatectomy or radiotherapy, without neoadjuvant hormone therapy.<br>TCGA, MSKCC, Baca, Berger, and Barbieri focused on localized prostate cancer, irrespective of risk group or age. Patients were treated with radical prostatectomy, with or without neoadjuvant hormone therapy.<br>EOPC (Gerhauser and Weischenfeldt) focused on early-onset prostate cancer (i.e. men <55 years of age). Patients were treated with radical prostatectomy, with or without neoadjuvant hormone therapy.<br><br>Quigley and Abida focused on mCRPC. Patients were hormone refractory and tissue was obtained from biopsy of various sites, including bone, liver, and other visceral metastases.
Recruitment	Patients were identified based on the availability of public molecular datasets. No additional filtering (in or out) was used to select patients, except where data were unavailable. With the exception of CPCG, all localized disease cohorts focused on patients treated with radical prostatectomy (rather than radiotherapy). These patients tend (on average) to be younger and more physically fit than patients who undergo radiotherapy, and thus we cannot exclude the possibility that restriction to a single treatment modality may bias the cohort to some extent.
Ethics oversight	Patients in the Canadian Prostate Cancer Genome Network (CPCG) cohort (Table S1; n = 385) were consented for whole-genome sequencing and other molecular analyses and for reporting of anonymized clinical data, with approval from local Research Ethics Boards (UHN #11-0024 and 06-0822; CHUQ 2012-913:H12-03-192). Patients in the Mt Sinai Hospital cohort ('Mt Sinai'; n = 47) were consented for molecular analysis and reporting of anonymized clinical data, with approval from the Research Ethics Board at Mt. Sinai Hospital and the Lunenfeld Research Institute (MSH REB #14-0211-E and University of Toronto REB #35275).

Note that full information on the approval of the study protocol must also be provided in the manuscript.
